# Gender differences in family meal frequency and their association with substance use and mental health among middle and high school students

**DOI:** 10.3389/fpubh.2023.1123396

**Published:** 2023-04-14

**Authors:** Ting Luo, Sharon E. Cummins, Shu-Hong Zhu

**Affiliations:** ^1^Moores Cancer Center, University of California, San Diego, La Jolla, CA, United States; ^2^Herbert Wertheim School of Public Health, University of California, San Diego, La Jolla, CA, United States

**Keywords:** family meal frequency, gender differences, family dynamics, substance use, mental health

## Abstract

**Background:**

Family meals are associated with adolescent health outcomes. Studies have reported that girls are less likely than boys to have dinner with their families.

**Purpose:**

This study examined gender differences in family meal frequency and the relationship between meal frequency and other health measures, using a large and representative sample of California middle and high school students.

**Methods:**

This study analyzed data from the 2019–2020 California Student Tobacco Survey (159,904 students in grades 8, 10, and 12). Dinner with the family 5–7 times per week was defined as high frequency. Students reported substance use (of tobacco, marijuana, and alcohol) and rated their mental health and happiness in their home life. All analyses were weighted to reflect the California student population.

**Results:**

Fewer than half (44.7%) of students reported a high frequency of family meals, with boys more likely than girls and those who identified their gender in another way the least likely to do so (48.3%, 42.2%, 34.0%, respectively). Gender differences persisted across demographics and the quality of family relationships, and were evident as early as eighth grade. Less frequent family meals were associated with poorer mental health (OR = 1.34, 95% CI: 1.29–1.40) and substance use (OR = 1.27, 95% CI: 1.21–1.32), controlling for the effects of demographics and family dynamics.

**Conclusion:**

Gender differences in family meal frequency emerge early in adolescence and persist across demographics and family relationships. Given that family meals play a protective role in an adolescent’s life, these gender differences are concerning.

## Introduction

Family meals provide opportunities for parents to connect with their children on a regular basis, communicate with them about daily activities, exchange social context, monitor children’s moods and behaviors, and set an example for them (1–5). Frequent family meals have been demonstrated to be associated with numerous benefits for adolescents, including better dietary intake (6, 7), better school performance (8), less substance use (9), fewer problematic behaviors (9), and fewer mental health issues (8, 9). Studies have also found that frequent meals are associated with stronger family relationships and communication between family members, leading to a happy home life (5, 8, 9). The value of family meals has been so well established that the National Center on Addiction and Substance Abuse in 2001 designated the fourth Monday in September as “Family Day—A Day to Eat Dinner with Your Children” (4).

A study of secondary-school students found that the frequency of family meals decreased by 3% from 1999 to 2010 (10). Moreover, the decrease varied by demographics. Frequent family meals decreased by 9.3% among the lowest-income students, whereas the highest-income students experienced a 5.1% increase over the same time period (10). The reasons for this difference are not completely known. Another notable discrepancy in mealtime frequency is by gender. In 1999, girls were slightly less likely to participate in family meals than boys (10). By 2010, the rate of participation in family meals for girls decreased by 5% while remaining constant for boys (10).

Several other studies have also reported on gender differences in family meal frequency, although it has not been the focus of the work (8–12). Where gender differences are observed, they are limited to those who identify as male or female. The pattern is consistent: boys are more likely to report having frequent family meals than girls of the same age (8, 12, 13). Proposed explanations for gender differences include the suggestion that girls are more likely than boys to be affected by family instabilities (8, 12, 14), such as family economic problems and parents’ negative feelings, and more likely to skip family meals because they are uncomfortable or as a way to control their weight (15–17).

This study focuses on the gender differences in family meal frequency, using a large population survey of middle and high school students in California. The study examines gender differences in three categories (male, female, and those who identify in another way) and investigates the relationship between family meal frequency and students’ mental health and substance use.

## Methods

### Study participants

This study presents data from the 2019–2020 California Student Tobacco Survey (CSTS), which used a two-stage cluster sampling design to obtain a representative sample of the state’s public school students (18). The survey was conducted among secondary schools (grades 8, 10, and 12). The school served as the primary sampling unit and the classroom served as the secondary sampling unit. For middle schools, a simple statewide random sampling approach was used. High schools were first stratified into 35 regions before being randomly sampled (12). The number of high schools selected from each region was determined by the region’s proportion of students in the state. Overall, 608 out of the 3,056 eligible schools were invited to participate, and 482 schools agreed to participate in the survey. A total of 369 schools (47 were middle schools and 322 were high schools) responded to the survey prior to the COVID-19 closures. The survey was fielded from September 2019 to March 2020. The survey was planned to end in April 2020 but ended in March 2020 instead because schools across the state began to close due to the COVID-19 pandemic. While closures occurred on different dates, most schools closed between March 13 to 18, 2020. The data from 27 schools were excluded because their response rate was below 40% due to the COVID-19 outbreak and thus considered unacceptable. The overall response rate among eligible students was 68.3%. The sampling design and response rates were taken into account in the analysis through proper weighting. Furthermore, a post-stratification adjustment was applied, with weighted totals by grade recalibrated to equal population totals of eligible students in the region for each grade, based on California Department of Education enrollment data (18). The survey, available in English and Spanish, was anonymous and administered online during class time. There were 162,675 students who took the survey, but 2,771 (or 1.7%) did not respond to either frequency of family meals or gender (the variables of interest) and were excluded from the analysis, leaving an effective sample size of 159,904. The study was approved by the University of California Human Research Protections Program, Institutional Review Board #170787.

### Measures

The CSTS was primarily designed to determine the prevalence of tobacco use in California’s population of secondary students, but it also included other questions of interest. Family meal frequency was measured by asking: “In a usual week, how many times do all of the people in your family who live with you eat dinner together?” with response options of 0, 1, 2, 3, 4, 5, 6, and 7+ times a week (7, 9, 15). For analysis, response options were classified into two categories: 0–4 times per week and 5–7 times per week, with the latter category considered frequent family meals (FFM). This single-item measurement and classification of family meals has been applied in many studies (7, 8, 10, 19, 20).

Gender was assessed by the question “How do you describe yourself?” Seven options were given: (1) Male, (2) Female, (3) Female-to-Male/Transgender Male/Trans Man, (4) Male-to-Female/Transgender Female/Trans Woman, (5) Genderqueer, neither exclusively male nor female, (6) Additional gender category or other, and (7) Choose not to disclose. For analysis, the options were classified as Male, Female, Identified in Another Way (comprised of options 3–6), and Declined to answer. It is noted that the measurement of gender in this study is limited by questions asked in the survey and we adopted students’ response to these questions as an approximation of their gender identity.

The study included other demographic factors that have been associated with FFM (1, 21): ethnicity/race, parental education, and being the youngest child in the home. Ethnicity was defined using two questions: “Are you of Spanish or Hispanic (Latino or Latina) origin?” with answer options of yes and no and “How do you describe yourself?” with multiple options for race. Answers were recoded to Non-Hispanic (NH) White, NH-Black, Hispanic, NH-Asian, NH-others (including NH-American Indian, NH-Native Hawaiian and Pacific Islander, and NH-other race), and NH-multiple race. Parental education was assessed with the question: “Do either of your parents have a college education?” with options of yes, no, and “I do not know.” The youngest child was measured with a yes or no to the question: “Are you the youngest person living in your house?”

The survey also included two measures of family dynamics. Home life was assessed with the statements “I have a happy home life” (22) and “I can talk about my problems with my family” (23). Response options of strongly agree and somewhat agree were recoded as Yes, while somewhat disagree and strongly disagree were recoded as No for the analyses.

Substance use in this study was defined as any use of tobacco products, alcohol, or marijuana in the past 30 days. The survey contains many detailed questions on substance use. For tobacco use behavior, the survey asked whether the student had used cigarettes, vapes (e-cigarettes), little cigars and cigarillos, big cigars, smokeless tobacco, and hookah. Participants were presented with images and descriptions of each product and were asked if they had ever used it. Those who reported ever using a product were further asked if they had used it in the past 30 days. For marijuana use behavior, the survey asked if they had: smoked, ate, drank, dabbed, vaped, or used marijuana in some other way. For those who had ever used marijuana, they were asked if they had used the product in the past 30 days. Alcohol use was assessed by two questions: whether they had ever used alcohol (even just a few sips of any alcoholic drink) and whether they had used it in the last 30 days. Students who used any of these products in the last 30 days were considered current substance users.

Mental health was measured by the question, “In general, how would you rate your mental health?” Poor mental health was defined as anyone who chose the responses of “fair” or “poor” from the five options (excellent, very good, good, fair, or poor) (24).

### Analysis

Descriptive statistics were used to describe family meal frequency by demographic characteristics and family dynamics. Multiple logistic regression analysis was used to further assess the relationship between gender and FFM, controlling for other factors. A separate logistic regression examined the relationships between FFM and mental health, and the relationship between FFM and substance use, while controlling for other factors. The estimates of rates and proportions were presented with their 95% confidence intervals. The results were weighted to account for the complex survey design to be representative of California students. SAS software 9.4 was used for the analyses.

## Results

A total of 159,904 students were included in the study. By gender, the percentages were 45.7% males, 48.8% females, 2.9% who identified their gender in another way, and 2.5% who declined to provide their gender. While fewer 8th graders (7.4% of the sample) participated than 10th (50.2%) and 12th graders (42.4%), the rates and proportions reported in the results section were all weighted to reflect the population of these students in California. The ethnic breakdown was 52.7% Hispanic, 20.3% NH-White, 12.4% NH-Asian, 2.7% NH-Black, 3.6% NH-others, and 8.3% NH-multiple race. Almost half of the students (45.5%) reported that at least one of their parents had a college degree, and nearly two-fifths (38.4%) were the youngest child at home.

Table 1 shows the percentage of frequent family meals by gender. Girls were significantly less likely to have FFM than boys, 42.1% vs. 48.3%. Those who identified their gender in another way were the least likely (34.0%) to have FFM. Of those who declined to provide their gender, 43.7% reported FFM, a rate similar to that for girls.

**Table 1:** Percentage of middle and high school students having frequent family meals, by gender.

	Sample size	Total	Male	Female	Identified in another way	Declined to answer
	*N*	% (95% CI)	% (95% CI)	% (95% CI)	% (95% CI)	% (95% CI)
Mean	159,904	44.7 (43.9–45.6)	48.3 (47.4–49.2)	42.1 (41.0–43.2)	34.0 (31.6–36.4)	43.7 (40.9–46.5)
**Grade**						
8	11,786	52.2 (50.2–54.2)	55.2 (53.3–57.2)	50.2 (47.5–52.9)	39.7 (34.1–45.4)	47.6 (40.5–54.7)
10	80,105	44.4 (43.7–45.1)	48.6 (47.8–49.5)	41.3 (40.4–42.2)	33.4 (30.9–35.9)	41.5 (39.2–43.9)
12	68,013	37.2 (36.5–38.0)	40.2 (39.3–41.1)	34.9 (34.0–35.7)	27.7 (25.1–30.4)	41.6 (38.5–44.7)
**Race**						
NH-White	32,431	47.5 (45.5–49.5)	50.5 (48.1–53.0)	45.2 (42.7–47.6)	38.9 (32.1–45.7)	46.1 (37.4–54.7)
NH-Black	4,323	35.2 (30.4–40.0)	40.5 (33.0–48.0)	30.9 (26.1–35.7)	25.5 (16.6–34.4)	35.4 (24.9–45.9)
Hispanic	83,886	42.8 (42.0–43.6)	46.3 (45.3–47.2)	40.3 (39.3–41.4)	33.0 (29.7–36.3)	39.7 (36.1–43.2)
NH-Asian	19,880	50.9 (48.5–53.2)	54.3 (51.6–57.0)	48.5 (46.0–51.0)	33.8 (28.2–39.5)	44.5 (38.3–50.7)
NH-Others	5,680	45.1 (42.4–47.8)	49.2 (45.7–52.7)	40.8 (36.9–44.8)	35.9 (26.3–45.6)	48.1 (41.8–54.5)
NH-Multiple	13,322	46.5 (44.4–48.5)	50.3 (47.5–53.0)	43.3 (40.7–45.9)	32.7 (25.6–39.8)	52.3 (43.3–61.4)
**Parental education**						
College degree	72,743	49.6 (48.4–50.8)	52.6 (51.3–53.9)	47.2 (45.6–48.8)	43.1 (38.5–47.7)	44.6 (39.4–49.8)
No college degree	65,490	38.9 (38.2–39.6)	43.0 (42.1–43.9)	36.3 (35.2–37.4)	24.2 (20.1–28.3)	34.5 (29.5–39.5)
I do not know	21,359	45.6 (44.2–47.1)	48.1 (46.1–50.0)	44.1 (41.6–46.5)	31.6 (28.0–35.3)	49.1 (45.0–53.2)
**Youngest child in the home**						
Yes	61,344	41.3 (40.2–42.4)	44.2 (42.9–45.6)	39.2 (37.8–40.6)	31.3 (27.8–34.8)	40.0 (35.4–44.6)
No	98,371	46.9 (45.9–47.8)	50.8 (49.8–51.9)	43.9 (42.7–45.1)	36.0 (32.5–39.4)	45.2 (41.8–48.5)

Table 1 also shows a consistent gender pattern for FFM across demographic variables. While FFM declined with age (52.2% of 8th graders vs. 44.4% of 10th graders vs. 37.2% of 12th graders, respectively), the gender pattern of boys being the most likely to have FFM and students who identified in another way being the least likely to have FFM is clear at each age.

Likewise, while there were differences in FFM across ethnicity/race, the pattern across genders within each ethnic/racial group is the same. As shown in Table 1, NH-White students and NH-Asian students were more likely to have FFM than Hispanic students, and Hispanic students were, in turn, more likely to have FFM than NH-Black students. However, in each of these groups more boys than girls reported FFM (although among NH-Black students, the gender difference failed to reach significance). Again, students who identified in another way were the least likely to have FFM; however, due to smaller sample sizes, the confidence intervals were too large to determine significance. A similar pattern was evident with parental education. Students who had at least one parent with a college education more often reported FFM compared to students with lower parental education (49.6% vs. 38.9%, respectively), but within each of these education groups, boys participated in FFM more often than girls (52.6% for boys vs. 47.2% for girls among higher parental education and 43.0% for boys vs. 36.3% for girls among lower parental education). Those identifying in another way had the lowest FFM rates, although again, not all these differences reached significance. Finally, the same gender pattern emerged regardless of whether the student was the youngest child in the home. Being the youngest child at home was related to lower FFM (41.3% vs. 46.9%). But in either case (youngest or not), boys reported more FFM than girls, who in turn reported more FFM than students who identified in another way.

To rule out the possibility that the gender difference in FFM is caused by girls and boys having a different quality of life with their family members, Table 2 presents the rates of FFM separately for those who reported having a happy home life versus those who did not. On average, 83.9% of students reported that they had a happy home life and 60.7% said they were able to talk to their family about problems. However, gender differences persisted across these dimensions: regardless of whether students had positive or negative views of their family dynamics, boys were more likely than girls to have FFM. For students identifying their gender in another way, the pattern of being the least likely to have FFM was clear both among students who rated their home life as happy and among those who said they could talk with their family about problems. However, among those who identified in another way and indicated not having a happy home life or family to talk to, there was no significant difference in their rate of FFM compared to girls in households similarly perceived as unhappy and unsupportive.

**Table 2:** Percentage of students having frequent family meals by family dynamics and gender.

Happy home life?	Yes (*N* = 133,291)	% (95% CI)	No (*N* = 25,899)	% (95% CI)
Male	63,753	50.4 (49.5–51.3)	9,012	31.7 (29.8–33.6)
Female	63,734	46.2 (45.1–47.3)	14,223	23.9 (22.5–25.4)
Identified in another way	3,007	38.6 (35.6–41.7)	1,517	24.6 (21.2–28.1)
Declined to answer	2,797	46.3 (43.4–49.3)	1,147	38.1 (33.0–43.1)
Can talk with family?	Yes (*N* = 95,960)		No (*N* = 63,157)	
Male	47,000	53.1 (52.0–54.2)	25,730	38.9 (37.6–40.3)
Female	44,685	50.2 (49.0–51.4)	33,244	31.4 (30.1–32.6)
Identified in another way	2,199	37.3 (33.9–40.7)	2,322	31.2 (27.7–34.7)
Declined to answer	2,076	49.4 (45.4–53.4)	1,861	37.8 (34.3–41.4)

Table 3 shows the multiple logistic regression results between FFM and gender, controlling for demographics and family dynamics (including all variables in Tables 1, 2). Compared to boys, girls were 15% less likely to have FFM (OR = 0.85). Students who identified their gender in another way were even less likely to report FFM (OR = 0.68). The multivariate analysis confirmed the drop in FFM over age. Tenth and 12th graders were less likely than 8th graders to have FFM (OR = 0.75 and OR = 0.55, respectively). Using NH-White students as the reference, NH-Black students were significantly less likely (OR = 0.63) and NH-Asian students were more likely (OR = 1.28) to participate in FFM. Students from other ethnic groups did not differ in FFM from NH-White students when controlling for other factors. Students with a college-educated parent were 36% more likely to indicate having FFM than those without. Students with younger siblings in the home were 33% more likely to report FFM than those who were the youngest (OR = 1.33). When controlling for other factors, family dynamics were strongly related to FFM. Compared to students with a happy home life, those who did not have a happy home life were 46% less likely to have FFM (OR = 0.54, 95% CI: 0.51–0.57), and compared to those who could talk openly with their families, those who could not talk openly were 40% less likely to have FFM (OR = 0.60, 95% CI: 0.57–0.62).

**Table 3:** Association of demographics and family dynamics on frequent family meals, multiple logistic regression modeling (*N* = 158,418).

Variables		OR (95% CI)
Gender	Male	Ref
	Female	0.85 (0.82–0.88)
	Identified in another way	0.68 (0.60–0.77)
	Declined to answer	0.97 (0.86–1.10)
Grade	8	Ref
	10	0.75 (0.70–0.80)
	12	0.55 (0.51–0.59)
Race	NH-White	Ref
	NH-Black	0.63 (0.50–0.78)
	Hispanic	0.96 (0.90–1.02)
	NH-Asian	1.28 (1.17–1.41)
	NH-Others	0.95 (0.84–1.07)
	NH-Multiple	0.96 (0.86–1.06)
Parental college education or higher	No	Ref
	Yes	1.36 (1.31–1.43)
	I do not know	1.11 (1.04–1.18)
Youngest child	Yes	Ref
	No	1.33 (1.28–1.38)
Happy home life	Yes	Ref
	No	0.54 (0.51–0.57)
Can talk with family	Yes	Ref
	No	0.60 (0.57–0.62)

**Figure 1:**
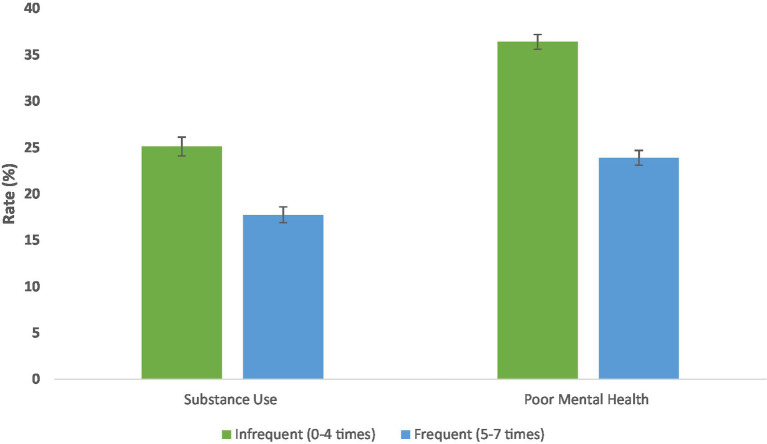
Percentage of substance use and poor mental health among adolescents with infrequent and frequent family meals.

Figure 1 shows that FFM predicts the likelihood of substance use for the study’s students. The rate of substance use was 25.1% for those who had meals with their family fewer than 5 times per week and 17.7% for those who had meals together at least 5 times per week. A multiple logistic regression model that controls for the effects of demographics and family dynamics (all variables shown in Table 3) confirms that this difference is statistically significant (OR = 1.27, 95% CI 1.21–1.32). In other words, students with low family meal frequency were 27% more likely to use substances than students who reported frequent family meals.

Figure 1 also shows that FFM predicts the mental health status of these students. The rate of poor mental health was 36.4% for those who had meals with their family fewer than 5 times per week versus 23.9% for those who had FFM. A multiple logistic regression model that controls for the effects of demographics and family dynamics (all variables shown in Table 3) confirms that this difference is statistically significant, with Odds Ratio = 1.34 (95% CI 1.29–1.40). In other words, students with low family meal frequency were 34% more likely to report poor mental health than students with FFM.

## Discussion

This study examined gender differences in the frequency of family meals among a representative sample of secondary students in California. Fewer than half of students reported FFM (at least 5 times/week). There were strong gender differences in FFM, with boys consistently reporting higher rates of FFM than girls, while students who identified their gender in another way had the lowest rates of FFM. Gender differences in FFM were already evident by middle school and were robust across demographic factors and family dynamics. This study also confirmed that frequent family meals were generally associated with better mental health and lower rates of substance use, controlling for all demographic and family-dynamic variables. Given the real-world consequences of poor mental health and substance use in adolescence, the persistent gender differences seen in the frequency of family meals warrant scrutiny.

The gender difference between boys and girls in FFM found in this study is consistent with previous findings both in direction and size (9, 10, 13). The most comparable study was that of Fulkerson et al., which used a large, nationally representative sample of 6th to 12th graders to examine participation in FFM (9). Although the study sample was primarily (86%) NH-White students and did not have gender classifications beyond male and female, it used the same FFM measure and definition as the current study (9). The focus of that work was the relationship of FFM to high-risk behaviors (e.g., tobacco, alcohol, and drug use) and the role of internal assets, such as achievement motivation, and external assets, such as family support (9). Of note, the Fulkerson et al. study found that male students were more likely to participate in FFM than female students (47.0% vs. 42.6%, respectively), a 4.4% difference (9). The study, in its focus on internal and external developmental assets, classified the gender difference as “slight” (9). The male and female difference found in this study was in the same direction and appeared to be larger, 6.2 percentage points. Sampling differences between these two studies prevent direct comparison, making it difficult to ascertain if 6.2% is indeed a larger difference than 4.2%. It is worth noting, however, that Fulkerson et al.’s study was based on a 2006 survey, while the present study was based on a 2019–20 survey. It is possible that the gap has widened over time, a trend that the Neumark-Sztainer et al. study (10) mentioned previously also suggests.

What is notable in the current study is how the gender differences persist across multiple demographic and family-dynamic variables. The differences are evident in middle school and expand throughout the high school years; they are persistent regardless of whether students reported having a good family relationship or not. Being a girl is associated with a lower frequency of family meals and represents an additional risk factor that can compound the effects of other demographic factors on substance use and poor mental health. The findings are even more profound for students who do not identify as male or female.

Gender differences may be underappreciated if they are attributed to factors that are less amenable to intervention. For example, boys may be hungrier during puberty due to hormones (25, 26) or girls may be more responsive to tensions in the home (8, 12, 14). While it is true that the trajectories for appetite suppressant hormones differ by gender, the differences in FFM emerge quite early making it unlikely to be the full story. Likewise, even if girls are more responsive to tensions in the home, this does not fully explain the gender differences in FFM seen in happy and supportive homes.

One possible explanation of why the gender differences are so pervasive is related more to the structure and expectations of family meal participation. For example, this study found that students who had younger siblings were more likely to participate in family meals. Gender at birth is not contingent on birth order. Among all genders, ethnicities, parental education levels, and family dynamics, some students have younger siblings and others do not. And yet, having younger siblings at home increases the likelihood of FFM. A child with younger siblings may benefit from the structure of family meals and participate simply because the meal is there. In homes with no young siblings, families may stop providing the safety net of family meals, undervaluing the many advantages of family meals apart from providing food. It makes sense that girls, who mature earlier than boys, might face this to an even greater degree. Girls may start eating at their friends’ homes or go out to eat. Parents focused primarily on whether the child is fed may not even be aware that family meals are decreasing.

Many studies have focused on the relationship between FFM and healthy eating patterns, and most of these have been conducted with girls (17, 27). Frequent family meals have been associated with a decrease in problematic eating behaviors such as extreme dietary restriction, anorexia and bulimia, skipping meals, overeating, and poor food choices (15, 28, 29). Interventions for families with overweight adolescent girls have had some success in increasing family meal participation by stressing its importance in their daughters’ weight loss efforts (30, 31). Yet this focus is perhaps too narrow and more reactive than would be ideal.

Most previous studies related to family meals have provided gender choices of male or female (7–10). By allowing students to identify as transgender, genderqueer, or in other terms, this study contributes to the literature on gender differences in family meals. Students who identified as other than male or female consistently had the lowest percentage of FFM. It is not clear what to make of this finding except to suggest that identifying in ways outside of the traditional male/female classification is associated with greater risk and merits further research. Recent work by VanKim and associates on gender expression and sexual orientation concluded from their analysis of the longitudinal Growing Up Today Study (1997–2011) that the relationship between sexual orientation, gender expression, diet quality, breakfast consumption, and family differences is complex (11).

### Limitations and strengths

The 2019–2020 California Student Tobacco Survey used a two-stage cluster sampling design to obtain data from a large, representative sample of secondary students and included an expansive definition of gender. The survey is representative of students in California, nearly 55% of whom identify as Hispanic, which may limit the generalizability of the findings to other populations. Also, the survey was developed primarily to provide stable prevalence estimates of tobacco use, not family meal frequency. As a result, additional factors relevant to FFM (e.g., family income, parental occupation and marital status, and attitudes and knowledge about the importance of family meals) were not measured. And because the survey was cross-sectional rather than longitudinal, no conclusions can be drawn about the causal nature of the relationships observed. Still, the careful sampling design, the large sample size (N > 159,000 students), and the multivariate analyses increase confidence in the findings.

## Conclusion

The current study with a large probability sample of students in California shows that frequent family meals are associated with less substance use and better mental health. It also suggests that families may be unaware of how the practice of having family meals together benefits adolescents, regardless of how mature they are perceived to be. And, finally, it confirms that there is a gender difference in family meals that starts early and can compound other risk factors for girls and students who identify their gender in ways other than male or female.

## Data availability statement

The raw data supporting the conclusions of this article will be made available by the authors, without undue reservation.

## Ethics statement

The studies involving human participants were reviewed and approved by University of California Human Research Protections Program, Institutional Review Board. Written informed consent from the participants’ legal guardian/next of kin was not required to participate in this study in accordance with the national legislation and the institutional requirements.

## Author contributions

TL: conceptualization, data curation, formal analysis, methodology, software, validation, visualization, writing original draft, and writing review and editing. SC: conceptualization, project administration, and writing review and editing. S-HZ: conceptualization, data curation, formal analysis, funding acquisition, investigation, methodology, project administration, resources, software, supervision, validation, and writing review and editing. All authors contributed to the article and approved the submitted version.

## Funding

This work was supported by a contract from the California Department of Public Health #CDPH-16-10109. The study sponsors had no involvement in (1) study design; (2) the collection, analysis, and interpretation of data; (3) the writing of the report; or (4) the decision to submit the manuscript for publication.

## Conflict of interest

The authors declare that the research was conducted in the absence of any commercial or financial relationships that could be construed as a potential conflict of interest.

## Publisher’s note

All claims expressed in this article are solely those of the authors and do not necessarily represent those of their affiliated organizations, or those of the publisher, the editors and the reviewers. Any product that may be evaluated in this article, or claim that may be made by its manufacturer, is not guaranteed or endorsed by the publisher.
